# Defining Gaslighting in Gender-Based Violence: A Mixed-Methods Systematic Review

**DOI:** 10.1177/15248380251344316

**Published:** 2025-07-12

**Authors:** Jewels Adair

**Affiliations:** 1 University of Windsor, ON, Canada

**Keywords:** gaslighting, gender-based violence, definition, manipulation, epistemic violence

## Abstract

In both public and academic discourse, gaslighting has gained increased attention, especially regarding psychological abuse, power imbalance, and gender-based violence (GBV). However, the term gaslighting is often inconsistently defined and conflated with broader forms of manipulation. It is also largely examined in the context of intimate partner violence (IPV), which ignores its occurrence in other forms of GBV. The present study presents a systematic review that synthesizes interdisciplinary academic literature to create a comprehensive framework of gaslighting. This framework includes the specific tactics that are used by perpetrators of gaslighting, the social–psychological outcomes experienced by survivors, and the role of systemic inequalities and social power dynamics. A search across multiple databases identified 96 records that discussed gaslighting in relation to GBV. Thematic analysis revealed a two-part framework for understanding gaslighting: (a) gaslighting tactics, which were categorized into cognitive and perceptual manipulation, emotional and psychological abuse, power dynamics and control, and additional forms of manipulation and (b) survivor outcomes, including disruptions to perception and memory, emotional distress, social isolation, and resistance strategies. The findings show that gaslighting is more than just an interpersonal act; it is sustained within social structures, where perpetrators use identity factors and forms of marginalization to exploit survivors. Overall, this review presents a comprehensive definition of gaslighting that illustrates its epistemic nature and its intersection with systemic oppression. It is suggested that future research studies gaslighting in GBV contexts beyond IPV, while practice and policy efforts should seek to enhance recognition and support for survivors.

The term “gaslighting” has recently become a large focus in both cultural and academic discussions, especially in regard to psychological abuse and interpersonal relationships (Carpenter, 2018; Sarkis, 2017; Stern, 2018). The American Psychological Association (APA) broadly defines gaslighting as the act of manipulating someone into doubting their own perceptions, experiences, or understanding of events (APA, 2023). The term gaslight originates from the play Gas Light by Hamilton (1938), which was then adapted for a film in 1944 (Cukor, 1944). This play is about a woman and an abusive husband who purposefully changes her physical environment to manipulate her and make her question her sanity. Although the concept was originally from this play in the 1940s, it was only recently that it became popular in both social discourse and research (Carpenter, 2018; Sarkis, 2017). For example, gaslighting became the Merriam-Webster word of the year in 2022 when searches for the term increased by 1,740% (Merriam-Webster, 2022).

Furthermore, while the term gaslighting has recently gained significant attention, its definition is still highly debated in both social and academic contexts (Hailes & Goodman, 2023; Paul-Mikhail, 2021; Spear, 2023). From a quick search online, you will find dozens of websites and checklists listing the “warning signs” of gaslighting. However, these checklists often explain general behaviors that occur in psychological abuse and do not explain the specific components that make gaslighting unique (e.g., Sarkis, 2017). Similarly, while the APA, as well as other sources, have created definitions of gaslighting, they are often not specific enough to distinguish it from other types of abuse (e.g., APA, 2023; Sarkis, 2017; Stern, 2018). There have also been several empirical studies that have proposed different definitions of gaslighting, but they tend to lack consistency and/or agreement on what should be included in the definition (Hailes & Goodman, 2023; Paul-Mikhail, 2021; Spear, 2023). For example, some of these studies explain that gaslighting is a form of epistemic violence, which can be defined as an attack on a victim’s^1^ ability to trust their knowledge and perceptions of the world (e.g., Field-Springer et al., 2021; Hailes & Goodman, 2023; Klein et al., 2023; Spear, 2020, 2023). In contrast, other studies explain that it is a type of psychological abuse (e.g., Sweet, 2019). Without a unified definition and rigorous empirical evidence to support it, the term gaslighting may risk becoming an overgeneralized term. Furthermore, since this term is continuing to gain popularity in both academic and social contexts, it is important to have definitional clarity to avoid it being dismissed as a buzzword rather than as a serious form of manipulation. Therefore, the present study seeks to clarify the complexities of gaslighting described in the literature, with the goal of developing an empirically grounded conceptual model for understanding this unique form of harm.

Although there is ongoing debate about the exact parameters of gaslighting and what should be included in its definition, recent work has highlighted the importance of a two-part conceptualization. In their article, Hailes and Goodman (2023) explain past findings on gaslighting and argue that it is best understood through both the tactics used by perpetrators and the outcomes experienced by victims. While tactics such as questioning someone’s perceptions or reality are commonly used in gaslighting, these actions alone do not constitute gaslighting unless they result in an impact on the victim, such as self-doubt (Hailes & Goodman, 2023). It’s necessary to include outcomes in a definition of gaslighting because it separates gaslighting from ways of responding, such as disagreement or dismissal. Furthermore, even though research has highlighted the range of tactics and outcomes that are involved in gaslighting (e.g., Hailes & Goodman, 2023; Klein et al., 2023; Sweet, 2019), there is still a need to synthesize these findings and establish a conceptual model of gaslighting.

Another issue with the current literature is that it tends to only study or frame gaslighting in the context of intimate partner violence (IPV; e.g., Bhatti et al., 2023; Hailes & Goodman, 2023; Sweet, 2019). In research on IPV, gaslighting is often framed as a tactic used by abusive partners to gain control (e.g., Graves & Samp, 2021; Hailes & Goodman, 2023; Sweet, 2019), though scholars have debated whether gaslighting requires conscious intent. As Spear (2020) noted, individuals may perpetrate gaslighting without self-awareness of their manipulative behavior. The problem with this focus on gaslighting occurring in IPV is that it overlooks the occurrence of gaslighting in other forms of gender-based violence (GBV), such as workplace harassment, sexual violence, and family violence (e.g., Field-Springer et al., 2021; Hershcovis et al., 2021; Newman et al., 2021). This pattern of only researching gaslighting that occurs in IPV is problematic because it silences the perspectives of individuals who experience gaslighting in other contexts. This systematic review aims to address this gap by examining how gaslighting is described across different types of GBV.

Furthermore, while gaslighting is commonly understood as an interpersonal dynamic, it would not exist without the systemic oppression and institutional structures that sustain it (Abramson, 2014; Graves & Samp, 2021; Klein et al., 2023; Sweet, 2019). By examining gaslighting through a social–psychological lens, research shows that it is not just a tactic used by an individual to manipulate their intimate partner but it is also a method of control used to maintain power imbalances (Sweet, 2019). Recent work has indicated that gaslighting often occurs within power-imbalanced relationships, and perpetrators of gaslighting systematically exploit individuals’ intersectional vulnerabilities and stereotypes about their identity (Sweet, 2019). Gaslighting often overlaps with systemic forms of oppression, such as sexism, racism, and heteronormativity, which amplifies its impact on marginalized individuals (Abramson, 2014; Graves & Samp, 2021; Hailes & Goodman, 2023; Klein et al., 2023; Sweet, 2019). Stereotypes rooted in gender, sexuality, race, class, and disability are often used as gaslighting tactics. For example, perpetrators often dismiss women’s voices and use gaslighting tactics by weaponizing sexist stereotypes, such as labeling women as “crazy,” “overly emotional,” or “liars” (Sweet, 2019). Individuals who weaponize gaslighting tactics may also exploit institutional systems to further harm victims. For instance, a perpetrator might threaten to call the police on a racialized individual because they know that systemic discrimination may work to their advantage (Sweet, 2019). When combined with other gaslighting tactics, such as blaming the violence on the victim, using institutions against the victim can escalate the harm if law enforcement sides with the perpetrator, which could result in the victim being arrested unfairly (Sweet, 2019). Overall, the literature suggests that gaslighting is a multifaceted social–psychological tactic of abuse with often devastating outcomes for survivors (e.g., Hailes & Goodman, 2023; Sweet, 2019). Therefore, it is imperative to develop a more nuanced understanding of how the existing literature defines, describes, and studies gaslighting.

## Current Study

To understand how gaslighting is being described and defined in the literature, one of the first goals of this systematic review was to explore the range of terms used to describe gaslighting within GBV. This was important because the language researchers use shapes how gaslighting is conceptualized. For instance, it is common for terms like “epistemic violence,” “psychological manipulation,” and “emotional abuse” to be used interchangeably with gaslighting. I specifically wanted to catalog the terms used synonymously or in relation to gaslighting, to reveal how gaslighting may be conflated with other concepts used to describe abuse or manipulation.

Next, to evaluate how gaslighting is defined in the literature, this systematic review adopted the two-part conceptualization that was recently proposed by Hailes and Goodman (2023). This conceptualization emphasizes examining both the tactics employed by perpetrators as well as the resulting outcomes experienced by survivors. Furthermore, since scholars argue that to understand gaslighting, it is important to examine how it is inherently both a social and psychological phenomenon (Abramson, 2014; Graves & Spencer, 2022; Sweet, 2019), I also assessed the broader social power dynamics and intersectional vulnerabilities that are involved in gaslighting as discussed in the literature. Therefore, to develop a more comprehensive and empirically grounded definition of gaslighting, I conducted a mixed-methods systematic review aimed to answer the following questions:

1. What terms are being used synonymously to describe gaslighting in relation to GBV?
2. How is gaslighting conceptualized and defined in the literature in relation to GBV, and what specific tactics used by perpetrators and outcomes experienced by victims are discussed in relation to these definitions?
3. How does the literature describe the role of social–psychological mechanisms in gaslighting within GBV, including the exploitation of individual vulnerabilities, stereotypes, and institutional structures? Additionally, how do social inequalities and power dynamics influence individuals’ vulnerability to gaslighting?

## Method

### Systematic Review Protocol

This review used a mixed methods approach to synthesize interdisciplinary literature that discussed gaslighting in relation to GBV. To conduct the analysis, I followed the guidelines of the 2020 Preferred Reporting Items for Systematic Reviews and Meta-Analyses (PRISMA), including the checklist for conducting systematic reviews to ensure comprehensive reporting of the findings (Page et al., 2021). The search terms were chosen from a review of key literature on gaslighting and through consultation with institutional reference librarians. The records included in this review reflect records published on or before September 30, 2024. The search parameters were not time-bound to allow for a comprehensive analysis of how gaslighting has been defined across any period in academic literature. The following search string was employed systemically for every database searched:

(“Gaslight*”) AND (“gender-based violence” OR “violence against women” OR “sexual assault” OR “sexual violence” OR “rape” OR “sexual harassment” OR “sexual abuse” OR “sexual victimization” OR “sexual trauma” OR “sexual coercion” OR “non-consensual sex” OR “alcohol facilitated rape” OR “forced sex” OR “sex trafficking” OR “sexual battery” OR “sexual exploitation” OR “unwanted sexual contact” OR “intimate partner violence” OR “domestic violence” OR “domestic abuse” OR “abuse”) AND (“term*” OR “definition” OR “define” OR “operationalize*” OR “describe” OR “concept” OR “notion”)

The following databases were searched: ProQuest Dissertation & Theses (*n* = 387), Scopus (*n* = 201), Sociological Abstracts & Applied Social Sciences Abstracts (*n* = 188), PsycArticle (*n* = 37), PsycINFO (*n* = 14), Academic Search Complete (*n* = 10), Medline (*n* = 5), Communication and Mass Media Complete (*n* = 2), JSTOR (*n* = 1). In addition, 22 records were also found from backward searching of key articles.

#### Eligibility Criteria

The articles that were chosen for this review included peer-reviewed articles, such as original empirical studies, commentaries, review papers, theory-building articles, and edited books. Furthermore, unpublished dissertations were included in this systematic review because gaslighting is a relatively new area of research, with most studies published within the last 5 years. Many dissertations in this field may not yet have had the opportunity to be published as journal articles, so excluding them could result in overlooking important insights. Including dissertations also aligns with systematic review guidelines; for example, the PRISMA 2020 statement acknowledges that systematic reviews can include various types of reports, such as journal articles, dissertations, and unpublished studies, depending on the scope and purpose of the review (Page et al., 2021). To maintain rigor, only dissertations with strong empirical methods and a clear definition of gaslighting were included. This meant that dissertations had to include an explicit definition of gaslighting that was either cited from an existing definition in the literature or they provided their own detailed definition. Dissertations that simply listed gaslighting tactics without providing a definition of gaslighting were not included. Non-academic articles, such as conference proceedings, policy bulletins, and magazines, were not included. Articles had to have been available in English and included a definition of gaslighting and/or an analysis of gaslighting in relation to GBV. Articles that did not have a translated version available in English were not included due to practical constraints. Given that this review was focused on examining definitions of gaslighting rather than the outcomes or findings of studies, a formal quality assessment of included records was not relevant to the aims of this review. All included records were scholarly sources, such as empirical studies, peer-reviewed theoretical and commentary articles, academic dissertations, and scholarly books, that met the criteria for the present study.

#### Screening Procedure

**Figure 1.**
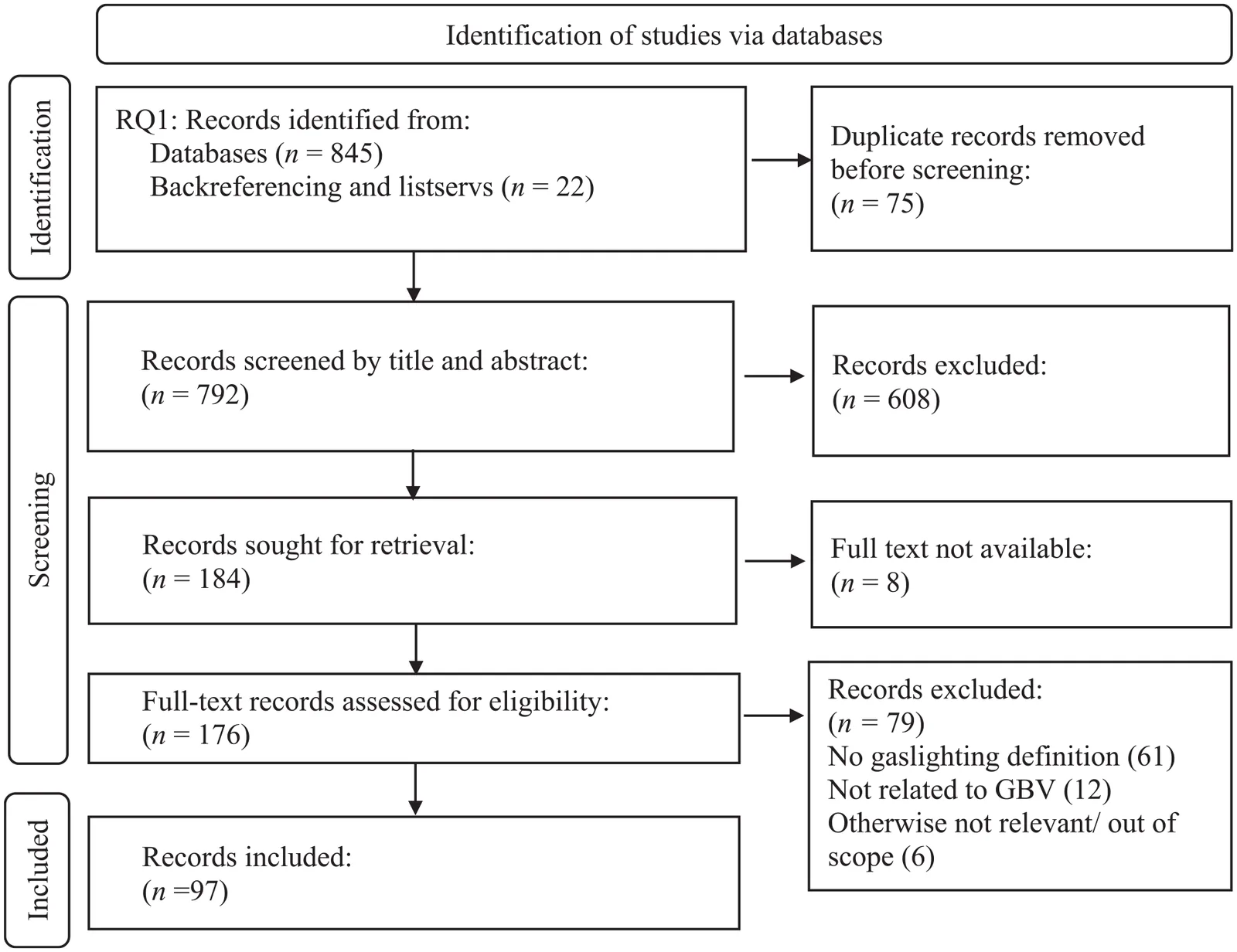
PRISMA flow diagram. *Source.* Page et al., (2021). *Note.* GBV = gender-based violence; PRISMA = Preferred Reporting Items for Systematic Reviews and Meta-Analyses.

The initial search produced a total of 845 records and 22 records from backreferencing. Following this, all records were imported into Mendeley Reference Manager and screened by myself. Duplicates were then removed, leaving 792 records. I then screened the titles and abstracts of the records for relevance, which then left 184 records. These records were carefully assessed and, in this phase, articles that did not discuss gaslighting in relation to GBV were removed. The final sample for this systematic review included 97 articles (see Figure 1 for the PRISMA flow diagram).

#### Data Extraction and Analysis

After the screening was complete, I extracted data using a chart to organize key variables: article title, publication year, the country where the research took place, terms used to describe gaslighting, gaslighting definitions and discussions, and findings related to gaslighting.

#### Quantitative Analysis

I then conducted a quantitative analysis using descriptive statistics, including counts and percentages, to systematically summarize key characteristics of the included studies, such as publication year, location, and the GBV contexts in which gaslighting was defined. Furthermore, after the qualitative analysis was complete, I also used descriptive statistics to count the gaslighting tactics and outcomes.

#### Qualitative Analysis

This review included both a content analysis and a thematic analysis of the data. For research question 1, I used a content analysis to assess the terms used to describe gaslighting. The content analysis involved creating codes derived directly from the exact terminology used in the records, then counting the frequency of these codes used by different records, and finally interpreting the results (Krippendorff, 2013).

Next, a thematic analysis was used to identify, analyze, and report themes within the rest of the data (Braun & Clarke, 2006, 2020). This method was used to understand the tactics that perpetrators of gaslighting use, as described by the records, the outcomes that victims of gaslighting face, gaslighting social power dynamics, and vulnerabilities to gaslighting. To complete this analysis, I first read the extracted data. Next, I identified key codes or concepts directly from the records. For example, if a record described gaslighting as an abusive tactic where the perpetrator manipulates the victim into believing they were the one cheating in the relationship rather than the perpetrator, this description was coded as “infidelity role reversal” (e.g., Hailes & Goodman, 2023; Knott, 2023). The third step involved searching for themes and subthemes, which involved comparing and analyzing the different codes to understand which codes were related and could be incorporated into a broader theme or subtheme. Going back to the first example, the code “infidelity role reversal” was then incorporated into the broader theme of “victim blaming and role reversal” in the third step, which captured the manipulative strategy of shifting blame and redefining roles to undermine the victim’s sense of reality. The thematic analysis utilized a combination of inductive and deductive methodologies. The inductive aspect involved generating codes directly from the specific terminology found in the records, allowing patterns to be generated from the data. The deductive aspect involved organizing these codes into broader themes that were informed by existing literature and previous research.

## Results

### Descriptive Statistics

The year of publication of the studies included ranged from 1969 to 2024. Most studies were published within the last 10 years (*n* = 86, 90%), highlighting the recency of the research in this area. Out of the 97 articles included, most studies were conducted in English-speaking western countries such as the United States (*n* = 58), the United Kingdom (*n* = 10), Australia (*n* = 9), and Canada (*n* = 5). The remainder of studies were conducted across diverse geographical locations including Italy (*n* = 3), Uganda (*n* = 2), Burkina Faso (*n* = 1), China (*n* = 1), Egypt (*n* = 1), Iceland (*n* = 1), India (*n* = 1), Montenegro (*n* = 1), the Netherlands (*n* = 1), Palestine (*n* = 1), Spain (*n* = 1), and Russia (*n* = 1). The majority of studies were published articles or books (*n* = 81) and some unpublished empirical dissertations (*n* = 16). Of all of the records, most were empirical studies (*n* = 63), followed by commentaries and review papers (*n* = 31) and edited books (*n* = 3). Of the 63 empirical studies, the majority were qualitative (*n* = 47), with some mixed methods (*n* = 10) and quantitative studies (*n* = 6).

A large proportion of articles defined gaslighting in relation to IPV (65%, *n* = 63). But there were other forms of GBV that were discussed in relation to gaslighting, including sexual assault disclosures that were manipulated through gaslighting by individuals other than the perpetrator (such as family, friends, etc.), and this included 12 articles (13%). Other forms of GBV discussed included sexual harassment (*n* = 6, 6%), sexism or sexist discrimination (*n* = 5, 5%), and general GBV that wasn’t specified in the article (*n* = 5, 5%). Less common forms of GBV included perpetrators who manipulated sexual assault victims through gaslighting either during the assault or after (*n* = 2), gender and/or sexual identity-based violence (*n* = 2), and obstetric violence (*n* = 2).

### Terms Used to Describe Gaslighting

The most common term used to explain or define gaslighting was manipulation (*n* = 30). Other terms included psychological abuse (*n* = 22), focusing on emotional and mental harm; abuse (*n* = 14), a general term for harmful behaviors; epistemic injustice (*n* = 12), which refers to the denial or undermining of someone’s ability to trust their knowledge, control or coercive control (*n* = 5), involving tactics to remove one’s agency, covert abuse (*n* = 4), meaning hidden or subtle harm, mind games (*n* = 3), referring to actions designed to confuse or destabilize the victim, and testimonial injustice (*n* = 3), which occurs when someone’s credibility is unfairly questioned. It is important to note that several recent studies have recommended framing gaslighting as a form of epistemic injustice because gaslighting fundamentally undermines the victim’s ability to trust their own knowledge, perceptions, and experiences (*n* = 12). A list of citations that used each term to describe gaslighting is provided in the supplemental materials.

### Tactics Used in Gaslighting

From the literature, the first theme I identified relating to the tactics used in gaslighting was cognitive and perceptual manipulation, which refers to tactics designed to distort the victim’s understanding of reality, memory, or perceptions. This theme included the tactics: reality and/or perceptual distortion (*n* = 74, 67%) and manipulating or misrepresenting a situation (*n* = 60, 62%). These tactics function to disorient the survivor, creating an environment where their sense of reality is persistently undermined. This theme also included denial and/or dismissing abuse (*n* = 58, 60%) and manipulating memory (*n* = 41, 42%), which were described as strategies used to invalidate the survivor’s experiences. Lastly, this category included minimizing, normalizing, and/or justifying abuse (*n* = 41, 42%).

The next theme, emotional and psychological manipulation, encompassed tactics targeting the survivor’s emotions and self-esteem. Within this theme, the most common tactic was making the victim feel crazy or calling them crazy (*n* = 56, 58%), which was described as an attempt to undermine their sanity. Another was attacking their confidence or belittlement (*n* = 43, 44%). Love bombing or manipulative charm (*n* = 15, 16%) was also discussed, highlighting how perpetrators use excessive affection to confuse and destabilize survivors.

The third theme identified was power dynamics and control, which captures how gaslighting is used to assert dominance over survivors. Tactics in this theme included exerting power and control (*n* = 37, 38%), isolating and alienating survivors from their support systems (*n* = 25, 26%), and exploiting social inequalities and stereotypes (*n* = 25, 26%) which underscore the role of power imbalances in facilitating gaslighting. Additionally, institutional manipulation and threatening (*n* = 16, 16%) were also included in this theme, which indicates how perpetrators exploit institutional systems against survivors, such as law enforcement, to maintain control. For example, a perpetrator might use a tactic like reversal of roles by telling a racially marginalized victim that they committed the abuse instead of the perpetrator, and then the perpetrator might escalate the effect of this tactic by threatening to call the police on them because they know that the victim is more likely to be unfairly targeted by law enforcement.

Finally, several tactics could not be grouped into broader themes and were categorized as distinct themes. These included victim blaming and role reversal (*n* = 45, 46%), long-term or repetitive manipulation (*n* = 19, 20%), and targeted aggression and/or intimidation (*n* = 13, 13%). A full list of the definitions of the qualitative tactics and quotes from the records can be found in Table 1. A list of citations that discussed each tactic is provided in the supplemental materials.

**Table 1.** Gaslighting Perpetrator Tactics.

Theme	Subtheme/tactic	Definition	Quote from record	*n*
Cognitive and perceptual manipulation	Reality and/or perceptual distortion	This tactic involves the manipulation of survivors’ perceptions and reality, often making them doubt their own perceptions, reality, and understanding of events.	“She started struggling with her perception of reality, of her own actions” (Zarr, 2021).	74
	Manipulating/misrepresenting a situation	This tactic covers deception and manipulation by perpetrators to create false narratives or distort situations.	“It refers to the emotional manipulation of the victim by the aggressor to convince her that she misunderstands situations, sowing doubts in her perception (Stern, 2018)” (Badenes-Sastre et al., 2023).	60
	Denial and/or dismissing abuse	This is the tactic where perpetrators refuse to acknowledge their abusive actions or dismiss an act of violence, thereby invalidating the survivor’s experiences.	““Gaslighting:” a type of psychological abuse where perpetrators make victims doubt themselves through purposeful denial and misinformation (Sweet, 2019)” (Han & Liu, 2023).	58
	Manipulating memory	This includes tactics where the perpetrator distorts or challenges the survivor’s memory to make them question their recollections of events.	“One of the most pervasive domains and one of the most objective was survivors’ memory. Harming partners challenged survivors’ memory by accusing them of things the survivors knew they had not done, like cheating or stealing” (Hailes & Goodman, 2023).	41
	Minimizing, normalizing, and/or justifying abuse	This tactic captures how perpetrators downplay the severity of their actions or rationalize them to make the survivor believe the abuse is normal or justified.	“About half (50.96%) of participants mentioned ‘trivializing,’ which involves making the victims believe their thoughts or identities are not important” (Li & Samp, 2023).	41
Emotional and psychological manipulation	Making the victim feel crazy or calling them crazy	This tactic highlights how perpetrators make survivors feel as if they are losing touch with reality, often accusing them of being mentally unstable or “crazy.”	“Gaslighting specifically involves making someone feel like they are crazy and cannot trust their own perceptions” (Graham, 2022).	56
	Attacking confidence/belittlement	This tactic relates to how perpetrators erode the survivor’s self-esteem by belittling their abilities and worth.	“As well as forms of gaslighting, such as questioning memory, refusing conversation, denying injustice, changing focus to question credibility and belittling or disregarding feelings” (Omran & Yousafzai, 2023).	43
	Love bombing/manipulative charm	This tactic captures the use of excessive affection and charm by perpetrators to confuse and control the survivor.	“For example, Sarkis (2018) suggests that relationships wherein gaslighting occurs begin with a period of ‘lovebombing’ that is characterized by excessive affection, attention, gifts, and charming behaviors.” (Klein et al., 2023).	15
Power dynamics and control	Exerting power and control	This tactic encompasses the use of gaslighting as a means to assert dominance over the survivor, particularly by exploiting power imbalances.	“Toward that end, we view gaslighting as a discursive dynamic drawing on reservoirs of social power to create language structures that destabilize a gaslightee’s sense of reality embedded in their knowledge claims” (Graves & Spencer, 2022).	37
	Isolation and alienation	This tactic reflects how perpetrators isolate survivors from their support systems, making them more dependent on the abuser.	“The perpetrator in psychological gaslighting is able to cause psychological breakdown by drawing upon a situation in which he can enlist features of their shared environment (that he has isolated her . . .)” (Pohlhaus, 2020).	25
	Inequality/stereotype exploitation	This tactic involves the use of social stereotypes and inequalities by perpetrators to undermine and discredit survivors, particularly those from marginalized groups.	“Rosalyn’s abuser also undermined her in front of police, relying on stereotypes about black women as aggressive” (Sweet, 2019).	25
	Institutional manipulation and threatening	This tactic includes threats and manipulation involving institutions, such as the legal system.	“Gaslighting relies on ‘structural and institutional inequalities’ (Sweet, 2019, p. 856), making vulnerable groups, often women,susceptible to victimization” (Jones, 2023).	16
Victim blaming and role reversal		This tactic involves perpetrators shifting the blame onto the victim, often reversing roles to portray themselves as the victim.	“Cecilia’s partner was so skilled at gaslighting that even when she found proof of her partner’s cheating, he justified it away . . . Ani was accused of cheating when it was actually her partner who was. She felt that she was in the wrong” (Knott, 2023).	45
Long-term/repetitive manipulation		This tactic refers to the continuous and persistent use of manipulative tactics over a prolonged period to wear down the survivor’s resistance.	“Constant barrages of abuse, accusations of getting it wrong” (Hayes & Jeffries, 2016).	19
Targeted aggression and/or intimidation		This tactic includes the targeted use of physical or verbal aggression to intimidate the survivor, often followed by attempts to distort the survivor’s perception of the event.	“One example of how aggressive gaslighting could be includes a participant who was physically attacked by her partner who claimed she had provoked him and was fearful of her.” (Arabi, 2022).	13

### Gaslighting Outcomes for Survivors

The first theme identified regarding the impact of gaslighting on survivors was cognitive and perceptual disruptions. Within this theme, the most commonly discussed outcomes found in the literature were disruptions to perceptions and/or reality (*n* = 54, 56%) and disruptions to memory (*n* = 33, 34%). Other outcomes included doubting their lived experiences (*n* = 23, 24%) and experiencing general confusion (*n* = 20, 21%). Findings from the literature highlight how survivors experience these disruptions, with many describing how gaslighting caused them to doubt their reality and memories.

The next theme, psychological and emotional impact, reflects the harmful effects of gaslighting on survivors’ mental health and well-being. This included self-doubt and/or loss of confidence (*n* = 50, 52%), which were frequently discussed in the literature, as were disruptions to sanity and/or feelings of instability (*n* = 45, 46%). The literature also reported that survivors of gaslighting often experience significant distress, including symptoms of anxiety and depression (*n* = 21, 22%), and many blamed themselves for the violence (*n* = 8, 8%).

The third theme identified was labeled as social and relational outcomes and highlights the broader social consequences of gaslighting. Survivors frequently experienced isolation (*n* = 18, 19%) and silencing (*n* = 12, 12%). The literature explained how perpetrators of gaslighting often use silencing tactics by making the survivor believe that others will neither understand nor believe their experiences.^2^

Finally, the last theme for survivor outcomes that was identified was resistance and coping mechanisms, which highlights the ways in which survivors attempted to navigate and counteract gaslighting. A small number of studies discussed instances where survivors demonstrated resistance or partial resistance to gaslighting (*n* = 8, 8%), while others described survivors using tools such as recording conversations to combat gaslighting (*n* = 4, 4%). These studies indicate that despite the manipulative tactics employed by perpetrators, some survivors were able to reclaim a sense of agency. A full list of the definitions of these qualitative outcome themes and quotes can be found in Table 2. A list of citations that discussed each gaslighting outcome is provided in the supplemental materials.

**Table 2.** Effects of Gaslighting on Survivors.

Theme	Subtheme/Outcome	Definition	Quote from record	*n*
Cognitive and perceptual disruptions	Disruptions to perceptions or reality	This is the impact of gaslighting tactics that lead survivors to question their own perceptions and reality.	“They were also subjected to gaslighting (‘Gaslighting,’ 2012), defined as a form of manipulation in which false information is presented to the victims, making them doubt their own memory, perceptions, and sanity” (Leedom et al., 2019).	54
	Disruptions to memory	This outcome highlights how gaslighting can cause survivors to question the accuracy of their memories.	“Indeed, others also alluded to their experiences of it and how they started to doubt their perceptions: . . . I ended up essentially a wreck, not trusting my own memory or interpretation of events” (Bates, 2020).	33
	Doubt their experiences	This outcome encompasses how gaslighting can cause survivors to doubt whether their experiences were real, leaving them unsure of what truly happened.	“In this regard, because of the gaslighting effect, victims of IPVAW may begin to doubt their experiences and feel powerless to leave violent relationships” (Badenes-Sastre et al., 2023).	23
	General confusion	This outcome describes the general state of confusion that gaslighting induces.	“Result of gaslighting tactics and behaviors causes the gaslightee to experience confusion, increasing self-doubt, and the urge to retreat” (Hightower, 2017).	20
Psychological and emotional impact	Self-doubt or loss of confidence	This outcome is the internal struggle survivors face as they begin to doubt themselves and lose confidence in their judgments and abilities.	“Survivors experienced varying degrees of self-doubt: on a continuum from complete self-doubt to complete resistance” (Hailes & Goodman, 2023).	50
	Disruptions to sanity or feeling unstable	This outcome describes the feeling of instability and questioning of one’s mental health state.	“The accusations, they nearly drove me insane, like to the point of where I nearly ended up in a psychiatric unit because I couldn’t—because he used to accuse me of all this stuff and I used to say I’m not doing it . . . (Kaitlyn)” (Tarzia & Hegarty, 2023).	45
	Distress, anxiety, depression, etc.	This outcome involves psychological distress, such as anxiety and depression, that survivors endure as a result of gaslighting.	“Mild depression and anxiety are now believed to be common symptoms for victims of gaslighting” (Gass & Nichols, 1988) (Dickson et al., 2023).	21
	Blamed themself for the violence	This outcome is the internalization of blame, where survivors start to believe that they were at fault for the violence they experienced.	“She started struggling with her perception of reality, of her own actions, she started to wonder, did he really push her? Or did she fall while he was trying to catch her? Maybe she was wrong. He seemed so certain” (Zarr, 2021).	8
Social and relational outcomes	Isolation	This outcome explains how survivors feel isolated from others, often believing that they are alone and unsupported.	“Finally, social isolation may have contributed to survivors’ sense of “losing their on reality” or becoming a “shell of themselves” (Klein et al., 2023).	18
	Silenced	This outcome involves the suppression of survivors’ voices, making them feel as though they cannot speak out about their experiences due to fear of disbelief or dismissal.	“Made to feel like those around them—indeed, those closest to them or those they would hope to trust—are unlikely to take their report seriously can perpetuate silence” (S. Stewart, 2019).	12
Resistance and coping mechanisms	Resistance or partial resistance	This can be explained as moments when survivors begin to resist gaslighting tactics.	“Some survivors pointed to specific turning point moments, when they began to resist (such as hearing them lie to a child)” (Hailes & Goodman, 2023).	8
	Needed to use tools to combat gaslighting	This involves the active steps survivors take, such as recording conversations, to protect themselves from further manipulation.	“I started recording our arguments because he keeps saying I’ve said things that I know I didn’t. Or that he didn’t say things I know he did. He has been away this weekend and it gave me time to listen to the recordings” (Leitão, 2021).	4

### Social Power and Intersectional Vulnerabilities to Gaslighting

To build on the findings above, which indicate that perpetrators use social–psychological tactics such as leveraging the fear of institutional repercussions and exploiting social inequalities, I sought to further explore the broader social dynamics involved in gaslighting. This led to the identification of a key theme: intersectional vulnerabilities that amplify an individual’s susceptibility to gaslighting. The records assessed in this review highlighted that intersectional marginalization not only increases an individual’s vulnerability to gaslighting but also intensifies its negative impacts, as perpetrators strategically exploit these social and institutional disparities. More specifically, this analysis found that the literature described that women were more susceptible to gaslighting and experienced more severe outcomes (*n* = 27, 28%), this was also shown for racially marginalized individuals (*n* = 18, 19%), individuals who have experienced past victimization (*n* = 18, 19%), and individuals who identified as LGBTQ+ (*n* = 6, 6%). It is important to note that while a small number of studies focused on men's experiences of gaslighting in IPV contexts (*n* = 2), the majority of the articles identified women as being disproportionately affected and more likely to be victims of gaslighting (*n* = 27).

## Discussion

The present systematic review sought to understand how gaslighting is described and defined in the literature within the context of GBV. By including 97 interdisciplinary research articles, books, and theoretical papers, this review develops an empirically grounded conceptual model for understanding gaslighting. Overall, the findings indicate that the majority of research on gaslighting has occurred in the last 10 years. This suggests that there is an increasing awareness of this form of violence as an important area to be studied. Furthermore, findings indicate that most of the literature on gaslighting was conducted in western contexts such as the United States and the United Kingdom, which suggests that there is a notable gap in research on gaslighting in more diverse cultural contexts and societies.

To establish a better understanding of gaslighting, this review sought to develop a definition that encompassed both the tactics employed by perpetrators and the outcomes experienced by victims. This method identified common gaslighting tactics, including reality distortion (e.g., Calef & Weinshel, 1981; Graves & Spencer, 2022; Hailes & Goodman, 2023), manipulating situations (e.g., Badenes-Sastre et al., 2023; Hightower, 2017), denying or dismissing abuse (e.g., Alvarez & Miller-Ott, 2022; Arabi, 2022; Knapp, 2019), and making the victim feel crazy (e.g., Antonioli, 2019; Monterrosa, 2023; Sweet, 2019). These tactics are powerful because they undermine the survivor’s ability to trust their perceptions and memories. This reinforces doubt and confusion for the survivor, making it difficult for them to understand what is real and what is not (Hailes & Goodman, 2023; Sweet, 2019). For example, tactics such as distorting the victim’s perceptions or manipulating their memory make it difficult for the survivor to understand if past abuse occurred, or if there is something wrong with their memory or perceptions (Hailes & Goodman, 2023; Li & Samp, 2023; Sweet, 2019). In the same manner, the minimization and normalization of abuse that perpetrators of gaslighting use serve to downplay the harm done in a way that leads victims to question whether anything ever really happened (Bhatti et al., 2023; Klein et al., 2023; Spear, 2023).

Gaslighting tactics have far-reaching repercussions. Oftentimes, these repercussions extend beyond the immediate experiences of gaslighting, such as questioning reality, perception, or memories (Abramson, 2014; Hailes & Goodman, 2023; Hightower, 2017; Sweet, 2019). The findings from this systematic review indicate that the literature frequently discusses the impact of gaslighting on an individual’s sanity and well-being in the long-term (e.g., Bates, 2020; Dandelet, 2021; Gass & Nichols, 1988). Often, victims experience self-doubt, which can lead them to question who they are and their self-worth (e.g., Bhatti et al., 2023; Klein et al., 2023; Zarr, 2021). This also may influence their decision to leave an abusive relationship. If the survivor questions their ability to discern reality and doesn’t believe in themselves to make decisions, they will be less likely to leave an abusive situation and more likely to believe the abusive person’s narrative (Badenes-Sastre et al., 2023; Hailes & Goodman, 2023; Hegel et al., 2023; Omran & Yousafzai, 2023). Furthermore, these tactics and the combination of experiences a victim has can lead to anxiety, depression, and other mental health concerns (Dickson et al., 2023; Dorpat, 1996; Hailes & Goodman, 2023; Hightower, 2017; Sweet, 2019).

In line with recent scholarship that emphasizes the role of power imbalances and social inequalities in exacerbating the effects of gaslighting (Sweet, 2019), this review explicitly focused on the social power dynamics involved in gaslighting. This led to the finding that the literature (and direct participant quotes from the literature) discussed how perpetrators of gaslighting often exploit vulnerabilities related to gender, race, past victimization, and marginalization (e.g., Sweet, 2019). Further, the literature also indicated that when perpetrators exploit an individual’s vulnerabilities, this can lead to more severe outcomes for the victim (Sweet, 2019). Overall, findings indicate that women and gender-diverse individuals are more likely to be targeted by gaslighting and experience more severe outcomes compared to men (e.g., Abramson, 2014; Badenes-Sastre et al., 2023; Sweet, 2019). This is likely due to power dynamics in society that position women and gender-diverse individuals as less credible or trustworthy (Sweet, 2019). Furthermore, an individual’s intersecting identities may compound the effects of gaslighting. Individuals who have different marginalized aspects of their identity, such as their race, socioeconomic status, and sexual orientation, may be more likely to receive skepticism or diminished credibility from other individuals in society who are not marginalized (Sweet, 2019). Perpetrators of gaslighting may purposefully exploit these intersecting vulnerabilities to further isolate and silence them. The literature and the findings from this review support this, which indicates that racially and sexually marginalized individuals are more vulnerable to gaslighting (e.g., Abramson, 2014; Dorri et al., 2023; Sweet, 2019).

This review also highlighted how the literature discussed that perpetrators of gaslighting often leverage the fear of institutional repercussions to further control and manipulate victims. For example, a perpetrator might blame the violence on the victim and then escalate the effect of this tactic by threatening to call the police on them because the perpetrator knows that the victim is more likely to be unfairly targeted or disbelieved by law enforcement (Sweet, 2019). When perpetrators exploit intersectional vulnerabilities, this creates an even more difficult environment for victims of gaslighting to leave (Sweet, 2019). Overall, these findings show that it is necessary for research to assess the broader social context in which gaslighting occurs, because understanding how power imbalances and social inequalities impact victims of gaslighting is crucial to develop effective interventions to support them (see Table 3 for a summary of critical findings).

**Table 3.** Summary of Critical Findings.

• Gaslighting occurs in various GBV contexts, including IPV, sexual assault, stalking, and human trafficking. • Perpetrators of gaslighting use a range of diverse tactics; the most common include reality distortion, manipulating situations, denying or dismissing abuse, and trying to make the victim feel crazy. • Gaslighting leads to severe psychological and social outcomes for survivors. Survivors may experience disruptions in their perceptions of reality, self-doubt, disruptions to their sanity, and other mental health challenges, underscoring the profound impact of gaslighting on well-being. • Gaslighting disproportionately impacts marginalized groups: Women, LGBTQ + individuals, and racially marginalized individuals are more vulnerable to gaslighting and experience more severe outcomes. • Gaslighting is not merely a psychological phenomenon but also a social one. Perpetrators often weaponize social inequalities, exploiting vulnerabilities related to gender, race, and other forms of marginalization to manipulate and control victims.

### A Comprehensive Definition of Gaslighting in GBV

The current systematic review has illuminated the complex and multifaceted nature of gaslighting occurring in GBV contexts, highlighting the need for a clear and comprehensive definition that accurately reflects its dynamics and impact. Drawing upon the insights gained from the 97 articles examined in this review, and based on the core principles I outline below, I propose a new comprehensive definition of gaslighting in GBV:

Gaslighting *vb*. is a specific type of manipulation of an individual’s perceptions, reality, and memory characterized by an epistemic attack that undermines their capacity to know and understand the world. Gaslighting can involve a variety of tactics, including cognitive, perceptual, and psychological manipulation, exerting dominance through isolation and exploitation, victim blaming and role reversal, long-term or repetitive manipulation, and targeted aggression, and results in disruptions to a survivor’s perceptions, reality, memory, and sanity, as well as social and relational challenges. While gaslighting often leads to disempowerment, some survivors may develop strategies to resist and/or navigate its effects. Gaslighting is deeply rooted in social inequalities, particularly those related to gender, race, socioeconomic status, and other marginalized identities. Its impact is most pronounced when perpetrators exploit these structural disparities and stereotypes within power-imbalanced interactions, reinforcing the survivor’s disorientation and amplifying their vulnerability within broader social and institutional contexts.

**Figure 2.**
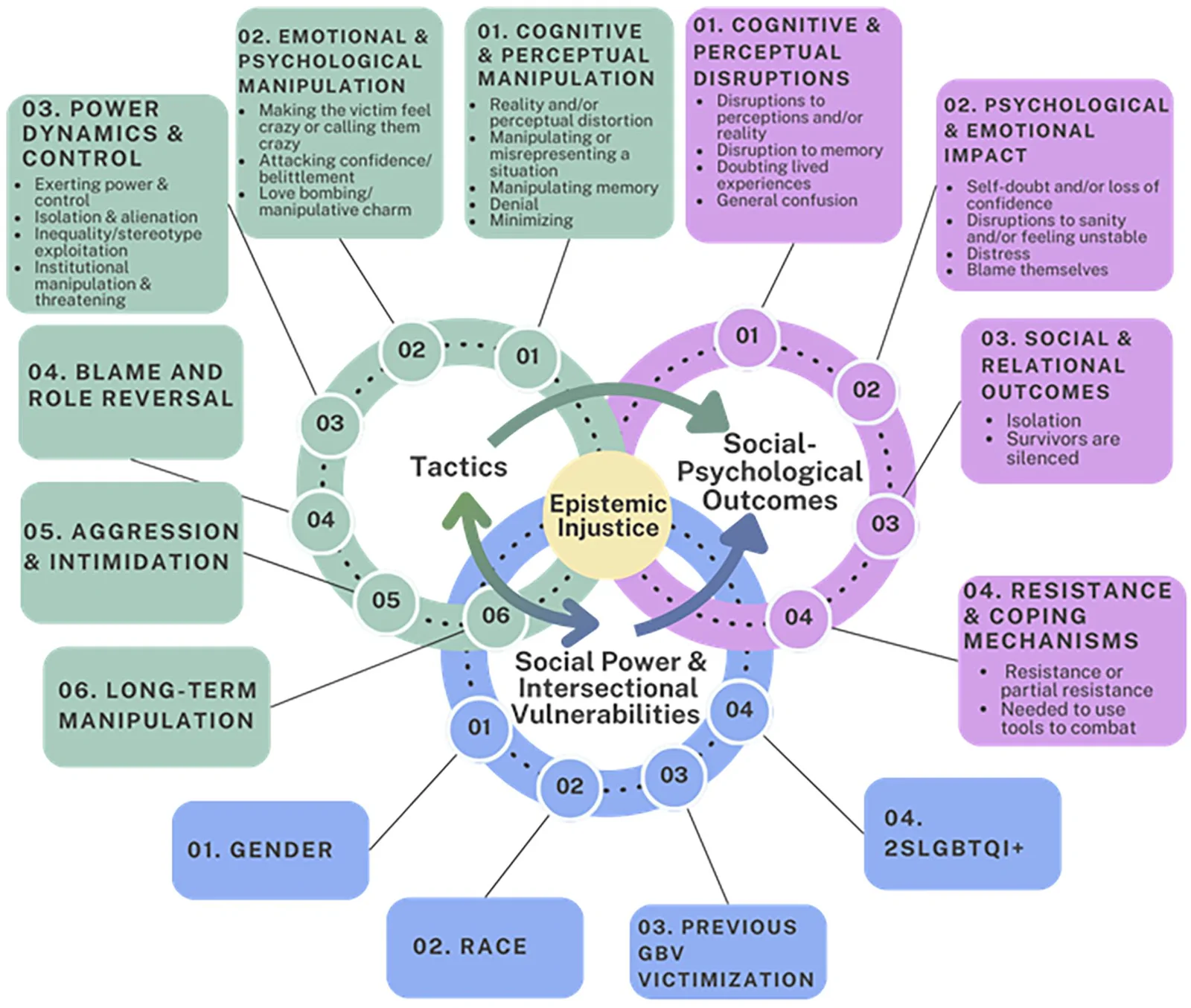
A conceptual model of gaslighting.

A pictorial representation of the definition can be found in Figure 2.

To develop this definition, I assessed the literature as well as the findings of this review to develop some guiding principles that are crucial for defining gaslighting. These principles emerged by integrating key insights from prominent researchers and theorists alongside my own observations of recurring patterns and themes in the findings of this review.

The development of this definition was guided by the first insight that emerged from this review: a comprehensive understanding of gaslighting must encompass the perpetrator’s tactics and the outcomes experienced by the victim (Hailes & Goodman, 2023). This principle emerged from suggestions in the literature that described how gaslighting is not simply a matter of disagreement; it involves manipulation that has consequences for the victim.

Secondly, within the literature and in popular discourse, gaslighting is often used interchangeably with other forms of manipulation. However, what differentiates gaslighting from other types of manipulation is its specific attack on an individual’s epistemic knowledge, or their capacity to know and understand the world (e.g., Field-Springer et al., 2021; Hailes & Goodman, 2023; Klein et al., 2023; Spear, 2020, 2023). The present review found that some of the most common gaslighting tactics discussed in the literature included reality and perception distortion, challenging a victim’s memory, and denying or dismissing abuse. These tactics all target and undermine the victim’s understanding of the world and themselves. Furthermore, perpetrators of gaslighting often directly attack a victim’s epistemic knowledge by accusing them of epistemic incompetence, for example, by labeling them as “crazy,” “emotional,” or “irrational.”

Finally, the question of intentionality in gaslighting is highly debated within the literature (Dickson et al., 2023; Spear, 2020). Many explain that it can be perpetrated with an unconscious awareness (e.g., Abramson, 2014; Spear, 2020), while others explicitly avoid attributing intentionality, instead framing gaslighting through its observable behaviors and outcomes (e.g., Hailes & Goodman, 2023; Sarkis, 2018). This lack of consensus highlights the need for future research to assess the role of intentionality in gaslighting. However, regardless of the perpetrator’s intent, the impact of gaslighting on victims remains significant. Therefore, the proposed definition that I’ve developed acknowledges this complexity by focusing on the observable behaviors and their consequences, rather than making assumptions about the perpetrator’s intentions.

### Implications for Research, Practice, and Policy

The findings from the present systematic review display how gaslighting is a psychological, social, and structural phenomenon within the context of GBV. These insights have important implications for research, practice, and policy. Overall, the majority of research on gaslighting has focused on its occurrence within IPV contexts, which has left a significant gap in our understanding of how gaslighting occurs in other forms of GBV. Future research should explore how gaslighting occurs in other types of GBV, such as sexual violence, harassment, stalking, and technology-facilitated GBV. Gaslighting may have similar dynamics and impacts when it’s used in other types of GBV, but because this has not yet been thoroughly researched, this is less clear. Additionally, there is a lack of studies that have assessed how marginalization and oppression are exploited by perpetrators of gaslighting, such as racism, sexism, and heteronormativity. Based on this gap, it is clear that research needs to incorporate intersectional approaches to studying gaslighting to understand how perpetrators weaponize multiple forms of marginalization that survivors experience, and how this may uniquely influence the outcomes that survivors have as a result of gaslighting.

Moreover, this review found that most of the studies on gaslighting were conducted in western, English-speaking countries, and mostly commonly in the United States. Future research should prioritize conducting studies in non-western and non-English-speaking countries and regions to understand potential cultural nuances in gaslighting or if there are no cultural differences, research should be clear about this and the extent to which findings can be generalized across different contexts.

While this review identified a range of gaslighting tactics and outcomes, future research should focus on assessing and refining these categories. For example, researchers could build on the themes identified in this review by developing and validating empirically based measurement tools that assess both the tactics used by perpetrators and the social–psychological outcomes that are experienced by survivors. These tools could then be applied across a variety of GBV contexts to compare the prevalence, dynamics, and impacts of gaslighting.

This systematic review highlights the importance for practitioners, specifically individuals who work in services related to mental health and GBV, to understand and incorporate a more nuanced understanding of gaslighting into their practice. It would be beneficial for practitioners to receive survivor-centered training that specifically teaches them how to recognize the specific gaslighting tactics and social–psychological outcomes that result from it. Furthermore, these approaches should also address the increased vulnerabilities that are faced by marginalized populations. This means that practitioners should be skillful at identifying gaslighting tactics, and it is important for them to understand how intersecting oppressions can compound the harm experienced by survivors.

For policy, the findings from this systematic review suggest that there is a need to incorporate a more comprehensive understanding of gaslighting into GBV prevention and response strategies. Policies should explicitly recognize gaslighting as a form of abuse within legal definitions of psychological violence and IPV. Training for law enforcement, legal professionals, and social workers must address the specific ways that gaslighting occurs, including the tactics that perpetrators use, the social–psychological outcomes experienced by survivors, and the social power dynamics that are involved. Furthermore, public awareness campaigns would also be useful tools to help clarify the definition of gaslighting and provide communities with knowledge that can help individuals recognize and challenge these abusive tactics (see Table 4 for a summary of implications).

**Table 4.** Summary of Implications for Future Research, Policy, and Practice.

• Research: Expand the study of gaslighting beyond IPV to include other forms of GBV. Prioritize intersectional research that explores how systemic oppressions are exploited by perpetrators of gaslighting and reinforced by social institutions. • Practice: Equip practitioners with training to recognize the nuanced tactics involved in gaslighting and its social–psychological outcomes. Emphasize the importance of cultural competence and trauma-informed approaches to address the compounded impact of social inequalities on marginalized survivors. • Policy: Integrate gaslighting into legal definitions of psychological abuse and IPV to ensure adequate protection for survivors. Develop training for legal professionals to improve responses to gaslighting in diverse contexts and implement public awareness campaigns to educate communities on recognizing and addressing these abusive tactics.

### Limitations

Although this systematic review provides valuable insights into the multifaceted nature of gaslighting within the context of GBV, several limitations should be acknowledged. First, the inclusion criteria for this review may have limited the analysis. While the inclusion criteria encompassed studies from any geographic region, the available literature reflects a significant research gap. Most studies originated from English-speaking western countries. This distribution represents a limitation not of the methodology of this review but of the research landscape, which has disproportionately focused on western contexts. Furthermore, this review focused exclusively on articles available in English, and this may have excluded research published in other languages. The dates that studies were published could be another limitation, even though this systemic review did not have time-based limits on what records were included. More specifically, the majority of the literature on gaslighting came from the past decade; therefore, this review is constrained by the recency of the research. This means that conclusions based on the findings from this review reflect a field in its early stages of development, and findings may evolve as the research expands and diversifies in the future. Additionally, consistent with this review’s focus on defining gaslighting and its social–psychological mechanisms, this scope necessarily excluded the examination of measures used to study gaslighting, or other factors related to gaslighting, such as survivor coping mechanisms. Future studies could expand beyond definition and conceptualization research to examine measures used to assess gaslighting and survivor coping methods.

## Conclusion

In conclusion, this systematic review provides a critical analysis of the existing literature on gaslighting definitions within the context of GBV. By emphasizing perpetrator tactics, survivor outcomes, and intersectional vulnerabilities from a social–psychological lens, this review provides a nuanced and empirically grounded definition and conceptual model for understanding gaslighting as a distinct form of manipulation. The findings highlight the importance of examining gaslighting across diverse GBV contexts using an intersectional analysis to understand how different individuals are harmed or exploited by perpetrators of gaslighting. Overall, this review lays the groundwork for future research to broaden the study of gaslighting across different contexts, develop measures, and explore targeted interventions.

## Supplemental Material

sj-docx-1-tva-10.1177_15248380251344316 – Supplemental material for Defining Gaslighting in Gender-Based Violence: A Mixed-Methods Systematic Review Supplemental material, sj-docx-1-tva-10.1177_15248380251344316 for Defining Gaslighting in Gender-Based Violence: A Mixed-Methods Systematic Review by Jewels Adair in Trauma, Violence, & Abuse

## Notes

1. This review uses the term victim/survivor interchangeably to (a) acknowledge the diverse ways individuals may identify and experience their journey during or after experiencing violence or trauma and (b) to align with the existing literature, which often uses these terms interchangeably.

2. It is important to note that while many of the tactics and outcomes listed here may occur in other types of abuse, they are included in this analysis because the reviewed literature specifically identified them as components of gaslighting. The outcomes listed were explained in the included records as being direct outcomes of gaslighting tactics, not of other forms of abuse. See Tables 1 and 2 for operational distinctions.
