# Supplementary material for: Defining Gaslighting in Gender-Based Violence: A Mixed-Methods Systematic Review
Source: Trauma Violence Abuse. 2025 Jul 12;27(4):1409–24. doi: 10.1177/15248380251344316 (PMC13578677; doi:10.1177/15248380251344316)
Supplement: Supplementary file 1 [file sj-docx-1-tva-10.1177_15248380251344316.docx]

**Supplementary Material: Complete List of Citations for Results Section**

**Supplemental Table 1.** Gaslighting Terms and Citations

| Term used to describe gaslighting | Citations that included the term |
| --- | --- |
| Manipulation | Abramson, 2014; Antonioli, 2019; Arabi, 2022; Badenes-Sastre et al., 2023; Bates, 2020; Bates & Carthy, 2020; Bhatti et al., 2023; Dickson et al., 2023; Dmitrieva & Glukhova, 2022; Dreke et al., 2020; Graham, 2022; Jeglic & Winters, 2023; Johnson et al., 2021; Knapp, 2019; Leedom et al., 2019; Leitão, 2021; Monterrosa, 2023; Namuggala & Oosterom, 2023; Nataraj-Hansen, 2023; Newman et al., 2021; Omran & Yousafzai, 2023; ; Paul-Mikhail, 2021; Pétursdóttir & Rúdólfsdóttir, 2022; Saltmarsh et al., 2022; Sarkis, 2018; Shousha, 2023; Spear, 2023; Stern, 2018; Sweet, 2019; Treviranus, 2021. |
| Psychological Abuse | Arabi, 2022; Dreke et al., 2020; Graves & Samp, 2021; Han & Liu, 2023; Hegel et al., 2023; Hightower, 2017; Khattar et al., 2023; Kim et al., 2023; Luxemburg-Peck, 2023; McKinnon, 2017; Merry, 2019; Mento et al., 2023; Miano et al., 2021; Monterrosa, 2023; Oliver, 2023; Omran & Yousafzai, 2023; Orzeck, 2008; Pickens, 2020; Shousha, 2023; Sweet, 2019; Wisdom & Nabors, 2024; Zarr, 2021. |
| Abuse | Dickson et al., 2023; Dorri et al., 2023; Haase & Worthington, 2023; Hailes & Goodman, 2023; Hightower, 2017; Klein et al., 2023; Merry, 2019; Omran & Yousafzai, 2023; Sarkis, 2018; Shousha, 2023; Spear, 2020; Stern, 2018; Sweet, 2019; Zarr, 2021. |
| Epistemic Injustice | Dandelet, 2021; Hailes & Goodman, 2023; H. Stewart, 2019a; Johnson et al., 2021; Luxemburg-Peck, 2023; McKinnon, 2017; Omran & Yousafzai, 2023; Paul-Mikhail, 2021; Pohlhaus, 2020; Spear, 2020; Spear, 2023; S. Stewart, 2019. |
| Coercive Control | Hailes & Goodman, 2023; Klein et al., 2023; Paul-Mikhail, 2021; Shousha, 2023; Graves & Samp, 2021. |
| Covert Abuse | Burnett, 2020; Dickson et al., 2023; Hightower, 2017; Zarr, 2021. |
| Mind Games | Hailes & Goodman, 2023; Pickens, 2020; Zarr, 2021. |
| Testimonial Injustice | McGlynn, 2020; Paul-Mikhail, 2021; Omran & Yousafzai, 2023. |

**Supplemental Table 2**. Gaslighting Tactics and Citations

| Gaslighting tactic | Citation that discussed it |
| --- | --- |
| Reality and/or perceptual distortion | Abramson, 2014; Alpert et al., 1998; Alvarez & Miller-Ott, 2022; Antonioli, 2019; Arabi & Ord, 2021; Badenes-Sastre et al., 2019; Bates, 2020; Bates & Carthy, 2020; Berenstain, 2020; Bhatti et al., 2023; Boxall, 2021; Calef & Weinshel, 1981; Dandelet, 2021; DeCrosta & Spear, 2024; Dickson et al., 2023; Dorpat, 1996; Dorri et al., 2023; Dreke et al., 2020; Field-Springer et al., 2021; Filippini, 2005; Graham, 2022; Graves & Spencer, 2022; Guerin & Ortolan, 2017; Hailes & Goodman, 2023; Harris, 2021; Hasse & Worthington, 2023; Hayes & Jeffries, 2016; Hegel et al., 2023; Hester et al., 2023; Hightower, 2017; Jeglic & Winters, 2023; Johnson et al., 2021; Jones, 2023; Khattar et al., 2023; Kim et al., 2023; Klein et al., 2023; Knapp, 2019; Knott, 2023; Kroll et al., 2022; Leedom et al., 2019; Leitão, 2021; Li & Samp, 2023; McGlynn, 2020; McKinnon, 2017; Mento et al., 2023; Merry, 2019; Miano et al., 2021; Monterrosa, 2023; Newman et al., 2021; Oliver, 2023; Omran & Yousafzai, 2023; Orzeck, 2008; Paul-Mikhail, 2021; Pickens, 2020; Pohlhaus, 2020; Pétursdóttir & Rúdólfsdóttir, 2022; Rodrigues et al., 2021; Ruiz, 2020; H. Stewart, 2019; S. Stewart, 2019a; S. Stewart, 2019b; Salzberger, 2022; Sarkis, 2018; Shane et al., 2019; Shousha, 2023; Spear, 2023; Stern, 2018; Su, 2022; Sweet, 2019; Tanczer et al., 2021; Tarzia & Hegarty, 2023; Tormoen, 2019; Wisdom & Nabors, 2024; Zarr, 2021. |
| Manipulating or misrepresenting a situation | Abramson, 2014; Alpert et al., 1998; Arabi, 2022; Badenes-Sastre et al., 2023; Bates, 2020; Bhatti et al., 2023; Boxall, 2021; Burnett, 2020; Calef & Weinshel, 1981; Dandelet, 2021; DeCrosta & Spear, 2024; Dickson et al., 2023; Dmitrieva & Glukhova, 2022; Dorpat, 1996; Dorri et al., 2023; Dreke et al., 2020; Field-Springer et al., 2021; Gass & Nichols, 1988; Graham, 2022; Graves & Samp, 2021; Graves & Spencer, 2022; Gutowski, 2012; H. Stewart, 2019a; Hailes & Goodman, 2023; Han & Liu, 2023; Hasse & Worthington, 2023; Hegel et al., 2023; Herschcovis et al., 2021; Hightower, 2017; Jeglic & Winters, 2023; Johnson et al., 2021; Jones, 2023; Khattar et al., 2023; Klein et al., 2023; Knapp, 2019; Knott, 2023; Leedom et al., 2019; Leitão, 2021; Mcglynn, 2020; Mento et al., 2023; Monterrosa, 2023; Nataraj-Hansen, 2023; Newman et al., 2021; Oliver, 2023; Omran & Yousafzai, 2023; Pohlhaus, 2020; Pétursdóttir & Rúdólfsdóttir, 2022; S. Stewart, 2019; Saltmarsh et al., 2022; Sarkis, 2018; Shousha, 2023; Spear, 2020; Spear, 2023; Stern, 2018; Sweet, 2019; Tarzia & Hegarty, 2023; Tormoen, 2019; Treviranus, 2021; Wisdom & Nabors, 2024; Zarr, 2021. |
| Denial and/or dismissing abuse | Abramson, 2014; Alpert et al., 1998; Alvarez & Miller-Ott, 2022; Arabi, 2022; Barton & Whitehead, 1969; Bates, 2020; Bates & Carthy, 2020; Bhatti et al., 2023; Boxall, 2021; Burnett, 2020; Calef & Weinshel, 1981; DeCrosta & Spear, 2024; Dickens, 2006; Dickson et al., 2023; Dorpat, 1996; Dorri et al., 2023; Dreke et al., 2020; Gass & Nichols, 1988; Graham, 2022; H. Stewart, 2019a; Hailes & Goodman, 2023; Han & Liu, 2023; Harris, 2021; Hayes & Jeffries, 2016; Hegel et al., 2023; Hightower, 2017; Johnson et al., 2021; Khattar et al., 2023; Kim et al., 2023; Knapp, 2019; Knott, 2023; Kroll et al., 2022; Leitão, 2021; Li & Samp, 2023; Luxemburg-Peck, 2023; Merry, 2019; Monterrosa, 2023; Newman et al., 2021; Oliver, 2023; Omran & Yousafzai, 2023; Peeren et al., 2024; Pemberton & Mulder, 2023; Pohlhaus, 2020; Rodrigues et al., 2021; S. Stewart, 2019; Sarkis, 2018; Shousha, 2023; Spear, 2020; Spear, 2023; Stern, 2018; Su, 2022; Sweet, 2019; Tanczer et al., 2021; Tarzia & Hegarty, 2023; Treviranus, 2021; Whiting et al., 2012; Wisdom & Nabors, 2024; Zarr, 2021. |
| Manipulating memory | Abramson, 2014; Antonioli, 2019; Arabi, 2022; Bailey, 2020; Barton & Whitehead, 1969; Bates, 2022; Bates & Carthy, 2020; Bhatti et al., 2023; Boxall, 2021; Calef & Weinshel, 1981; Dickson et al., 2023; Dorri et al., 2023; Dreke et al., 2020; Graves & Spencer, 2022; Hailes & Goodman, 2023; Harris, 2021; Hayes & Jeffries, 2016; Hightower, 2017; Jones, 2023; Khattar et al., 2023; Kim et al., 2023; Klein et al., 2023; Leedom et al., 2019; Li & Samp, 2023; McKinnon, 2017; Mcglynn, 2020; Newman et al., 2021; Oliver, 2023; Omran & Yousafzai, 2023; Orzeck, 2008; Pickens, 2020; Ridout, 2020; Sarkis, 2018; Shousha, 2023; Spear, 2020; Spear, 2023; Stern, 2018; Sweet, 2019; Tarzia & Hegarty, 2023; Tormoen, 2019; Wisdom & Nabors, 2024. |
| Minimizing, normalizing, and/or justifying abuse | Abramson, 2014; Ahmad & Monterrosa, 2018; Alvarez & Miller-Ott, 2022; Bhatti et al., 2023; Boxall, 2021; Burnett, 2020; DeCrosta & Spear, 2024; Dickson et al., 2023; Dorpat, 1996; Dorri et al., 2023; Dreke et al., 2020; Gass & Nichols, 1988; H. Stewart, 2019b; H. Stewart, 2019a; Haase & Worthington, 2023; Hailes & Goodman, 2023; Harris, 2021; Hightower, 2017; Klein et al., 2023; Knapp, 2019; Knott, 2023; Li & Samp, 2023; McGlynn, 2020; Newman et al., 2021; Oliver, 2023; Omran & Yousafzai, 2023; Peeren et al., 2024; Pohlhaus, 2020; Rathus et al., 2019; Rodrigues et al., 2021; S. Stewart, 2019; Sarkis, 2018; Shousha, 2023; Spear, 2020; Spear, 2023; Stern, 2018; Su, 2022; Tormoen, 2019; Treviranus, 2021; Wisdom & Nabors, 2024; Zarr, 2021. |
| Making the victim feel crazy or calling them crazy | Abramson, 2014; Antonioli, 2019; Arabi, 2022; Barton & Whitehead, 1969; Bates, 2020; Berenstain, 2020; Boxall, 2021; Burnett, 2020; Calef & Weinshel, 1981; Dickson et al., 2023; Dorri et al., 2023; Dreke et al., 2020; Filippini, 2005; Gass & Nichols, 1988; Graham, 2022; Guerin & Ortolan, 2017; H. Stewart, 2019a; Hailes & Goodman, 2023; Harris, 2021; Hayes & Jeffries, 2016; Hegel et al., 2023; Hester et al., 2023; Hightower, 2017; Hueerta & Summers, 2022; Jeglic & Winters, 2023; Johnson et al., 2021; Jones, 2023; Klein et al., 2023; Knott, 2023; Leedom et al., 2019; Leitão, 2021; McKinnon, 2017; Mcglynn, 2020; Mento et al., 2023; Merry, 2019; Monterrosa, 2023; Muray, 2020; Nataraj-Hansen, 2023; Oliver, 2023; Omran & Yousafzai, 2023; Orzeck, 2008; Paul-Mikhail, 2021; Rathus et al., 2019; Saltmarsh et al., 2022; Sarkis, 2018; Shousha, 2023; Spear, 2020; Spear, 2023; Stern, 2018; Sweet, 2019; Tanczer et al., 2021; Tarzia & Hegarty, 2023; Tormoen, 2019; Whiting et al., 2012; Wisdom & Nabors, 2024; Zarr, 2021. |
| Attacking confidence/ belittlement | Abramson, 2014; Alvarez & Miller-Ott, 2022; Barton & Whitehead, 1969; Bhatti et al., 2023; Burnett, 2020; Dickson et al., 2023; Dmitrieva & Glukhova, 2022; Dorpat, 1996; Dorri et al., 2023; Gass & Nichols, 1988; Graves & Spencer, 2022; Hailes & Goodman, 2023; Hayes & Jeffries, 2016; Hightower, 2017; Jeglic & Winters, 2023; Johnson et al., 2021; Jones, 2023; Khattar et al., 2023; Klein et al., 2023; Knott, 2023; Leitão, 2021; Mento et al., 2023; Merry, 2019; Newman et al., 2021; Oliver, 2023; Omran & Yousafzai, 2023; Orzeck, 2008; Paul-Mikhail, 2021; Peeren et al., 2024; Pickens & Kadieva; Rodrigues et al., 2021; Ruiz, 2020; Saltmarsh et al., 2022; Sarkis, 2018; H. Stewart, 2019a; Shousha, 2023; Spear, 2020; Spear, 2023; Stern, 2018; Su, 2022; Sweet, 2019; Wisdom & Nabors, 2024; Zarr, 2021. |
| Love bombing/ manipulative charm | Arabi, 2022; Bhatti et al., 2023; Boxall, 2021; Hailes & Goodman, 2023; Hayes & Jeffries, 2016; Hightower, 2017; Jones, 2023; Klein et al., 2023; Li & Samp, 2023; Miano et al., 2021; Newman et al., 2021; Pohlhaus, 2020; Stern, 2018; Tarzia & Hegarty, 2021; Tormoen, 2019. |
| Exerting power and control | Arabi, 2022; Badenes-Sastre et al., 2023; Bates, 2020; Burnett, 2020; Dickens, 2006; Dickson et al., 2023; Dmitrieva & Glukhova, 2022; Dorpat, 1996; Dorri et al., 2023; Graves & Samp, 2021; Haase & Worthington, 2023; Hailes & Goodman, 2023; Harris, 2021; Hegel et al., 2023; Hightower, 2017; Jeglic & Winters, 2023; Johnson et al., 2021; Jones, 2023; Khattar et al., 2023; Klein et al., 2023; Knapp, 2019; Li & Samp, 2023; McGlynn, 2020; Miano et al., 2021; Oliver, 2023; Omran & Yousafzai, 2023; Paul-Mikhail, 2021; Pohlhaus, 2020; Ruiz, 2020; Ruiz & Berenstain, 2018; Sarkis, 2018; Spear, 2020; Spear, 2023; Stern, 2018; Sweet, 2019; Wisdom & Nabors, 2024; Zarr, 2021. |
| Isolation and alienation | Abramson, 2014; Barton & Whitehead, 1969; Bates, 2020; Bhatti et al., 2023; Boxall, 2021; Calef & Weinshel, 1981; Dandelet, 2021; Graham, 2022; Guerin & Ortolan, 2017; Hailes & Goodman, 2023; Hasse & Worthington, 2023; Hershcovis et al., 2021; Klein et al., 2023; Knott, 2023; Oliver, 2023; Omran & Yousafzai, 2023; Pohlhaus, 2020; Ridout, 2020; Sarkis, 2018; Spear, 2020; Stern, 2018; Sweet, 2019; Tarzia & Hegarty, 2023; Tormoen, 2019; Treviranus, 2021. |
| Inequality/ stereotype exploitation | Badenes-Sastre et al., 2023; Bates, 2020; Dickson et al., 2023; Dorri et al., 2023; Field-Springer et al., 2021; Gass & Nichols, 1988; Hailes & Goodman, 2023; Han & Liu, 2023; Harris, 2021; Johnson et al., 2021; Jones, 2023; Klein et al., 2023; Li & Samp, 2023; McGlynn, 2020; McKinnon, 2017; Merry, 2019; Oliver, 2023; Omran & Yousafzai, 2023; Paul-Mikhail, 2021; Peeren et al., 2024; Pickens, 2020; Ruiz & Berenstain, 2018; Shousha, 2023; Sweet, 2019; Treviranus, 2021. |
| Institutional manipulation & threating | Dickson et al., 2023; Dorri et al., 2023; Hailes & Goodman, 2023; Hightower, 2017; Jones, 2023; Kurpuppu et al., 2023; Oliver, 2023; Omran & Yousafzai, 2023; Pemberton & Mulder, 2023; Pohlhaus, 2020; Ruiz, 2020; Ruiz & Berenstain, 2018; Saltmarsh et al., 2022; Sweet, 2019; Tormoen, 2019; Treviranus, 2021. |
| Victim blaming and role reversal | Abramson, 2014; Alpert et al., 1998; Arabi, 2022; Bhatti et al., 2023; Boxall, 2021; Burnett, 2020; Calef & Weinshel, 1981; Davies & Kelly, 2023; Dickens, 2006; Dickson et al., 2023; Dorpat, 1996; Dreke et al., 2020; Field-Springer et al., 2021; Gass & Nichols, 1988; Graham, 2022; H. Stewart, 2019a; Haase & Worthington, 2023; Hailes & Goodman, 2023; Harris, 2021; Hayes & Jeffries, 2016; Hegel et al., 2023; Hightower, 2017; Jones, 2023; Khattar et al., 2023; Kim et al., 2023; Klein et al., 2023; Knapp, 2019; Knott, 2023; Leedom et al., 2019; Monterrosa, 2023; Nataraj-Hansen, 2023; Oliver, 2023; Omran & Yousafzai, 2023; Paul-Mikhail, 2021; Peeren et al., 2024; S. Stewart, 2019; Sarkis, 2018; Shousha, 2023; Spear, 2020; Stern, 2018; Su, 2022; Sweet, 2019; Tarzia & Hegarty, 2023; Treviranus, 2021; Zarr, 2021. |
| Long term/  repetitive manipulation | Abramson, 2014; Bates, 2020; Dickson et al., 2023; Graham, 2022; Hailes & Goodman, 2023; Hayes & Jeffries, 2016; Hegel et al., 2023; Hightower, 2017; Jeglic & Winters, 2023; Johnson et al., 2021; Jones, 2023; Knapp, 2019; Knott, 2023; Merry, 2019; Sarkis, 2018; Spear, 2020; Stern, 2018; Tarzia & Hegarty, 2023; Zarr, 2021. |
| Aggression and/or intimidation | Arabi, 2022; Boxall, 2021; Dickson et al., 2023; Hightower, 2017; Jeglic & Winters, 2023; Khattar et al., 2023; Li & Samp, 2023; Mento et al., 2023; Miano et al., 2021; Monterrosa, 2023; Sarkis, 2018; Stern, 2018; Sweet, 2019. |

**Supplemental Table 3.** Effects of Gaslighting on Survivors and Citations

| Social-psychological outcome | Citation that discussed it |
| --- | --- |
| Disruptions to perceptions or reality | Abramson, 2014; Bates, 2020; Bates & Carthy, 2020; Bhatti et al., 2023; Boxall, 2021; Burnett, 2020; Calef & Weinshel, 1981; Dandelet, 2021; Dickson et al., 2023; Dorpat, 1996; Dorri et al., 2023; Fielding-Singh & Dmowska, 2022; Gass & Nichols, 1988; Graham, 2022; Graves & Samp, 2021; Graves & Spencer, 2022; H. Stewart, 2019a; H. Stewart, 2019b; Hailes & Goodman, 2023; Harris, 2021; Hegel et al., 2023; Hester et al., 2023; Hightower, 2017; Jeglic & Winters, 2023; Johnson et al., 2021; Jones, 2023; Khattar et al., 2023; Klein et al., 2023; Knapp, 2019; Knott, 2023; Leedom et al., 2019; Leitão, 2021; Li & Samp, 2023; Mento et al., 2023; Merry, 2019; Monterrosa, 2023; Nataraj-Hansen, 2023; Newman et al., 2021; Oliver, 2023; Omran & Yousafzai, 2023; Orzeck, 2008; Paul-Mikhail, 2021; Pickens, 2020; Pétursdóttir & Rúdólfsdóttir, 2022; S. Stewart, 2019; Sarkis, 2018; Shousha, 2023; Spear, 2023; Stern, 2018; Su, 2022; Sweet, 2019; Tanczer et al., 2021; Tarzia & Hegarty, 2023; Zarr, 2021. |
| Disruption to memory | Abramson, 2014; Bailey, 2020; Bates, 2020; Bates & Carthy, 2020; Boxall, 2021; Burnett, 2020; Calef & Weinshel, 1981; Dandelet, 2021; Dickson et al., 2023; Graves & Samp, 2021; Graves & Spencer, 2022; Hailes & Goodman, 2023; Harris, 2021; Hayes & Jeffries, 2016; Jones, 2023; Khattar et al., 2023; Klein et al., 2023; Leedom et al., 2019; Leitão, 2021; Li & Samp, 2023; Newman et al., 2021; Omran & Yousafzai, 2023; Orzeck, 2008; Pickens, 2020; Ridout, 2020; Sarkis, 2018; Shousha, 2023; Spear, 2020; Spear, 2023; Stern, 2018; Tanczer et al., 2021; Tarzia & Hegarty, 2023; Tormoen, 2019. |
| Doubt their experiences | Abramson, 2014; Bates, 2020; Boxall, 2021; Calef & Weinshel, 1981; Dickson et al., 2023; Gass & Nichols, 1988; H. Stewart, 2019a; Hailes & Goodman, 2023; Han & Liu, 2023; Harris, 2021; Hershcovis et al., 2021; Hightower, 2017; Klein et al., 2023; Knott, 2023; Oliver, 2023; Omran & Yousafzai, 2023; Pemberton & Mulder, 2023; Pétursdóttir & Rúdólfsdóttir, 2022; S. Stewart, 2019; Spear, 2020; Stern, 2018; Treviranus, 2021; Zarr, 2021. |
| General confusion | Bailey, 2020; Bates, 2020; Boxall, 2021; Calef & Weinshel, 1981; Filippini, 2005; Gass & Nichols, 1988; Graham, 2022; Hegel et al., 2023; Hightower, 2017; Jones, 2023; Klein et al., 2023; Knott & Verginelli, 2023; Matheson et al., 2023; Mento et al., 2023; Newman et al., 2021; Omran & Yousafzai, 2023; Shousha, 2023; Stern, 2018; Sweet, 2019; Tormoen, 2019. |
| Self-doubt or loss of confidence | Abramson, 2014; Alvarez & Miller-Ott, 2022; Badenes-Sastre et al., 2023; Bates, 2020; Bhatti et al., 2023; Burnett, 2020; Calef & Weinshel, 1981; Dandelet, 2021; Dickson et al., 2023; Dmitrieva & Glukhova, 2022; Dorpat, 1996; Dreke et al., 2020; Gass & Nichols, 1988; Graham, 2022; Graves & Samp, 2021; H. Stewart, 2019b; Hailes & Goodman, 2023; Harris, 2021; Hayes & Jeffries, 2016; Hegel et al., 2023; Hightower, 2017; Jeglic & Winters, 2023; Johnson et al., 2021; Khattar et al., 2023; Klein et al., 2023; Knott, 2023; Leitão, 2021; Li & Samp, 2023; Matheson et al., 2023; Mento et al., 2023; Namuggala & Oosterom, 2023; Newman et al., 2021; Oliver, 2023; Omran & Yousafzai, 2023; Paul-Mikhail, 2021; Peeren et al., 2024; Pemberton & Mulder, 2023; Pohlhaus, 2020; Pétursdóttir & Rúdólfsdóttir, 2022; Rodrigues et al., 2021; Ruiz, 2020; S. Stewart, 2019; Sarkis, 2018; Spear, 2020; Spear, 2023; Stern, 2018; Sweet, 2019; Treviranus, 2021; Treviranus, 2021; Zarr, 2021. |
| Disruptions to sanity or feeling unstable | Abramson, 2014; Arabi, 2022; Barton & Whitehead, 1969; Bates, 2020; Boxall, 2021; Calef & Weinshel, 1981; Dandelet, 2021; Dickson et al., 2023; Dorpat, 1996; Dorri et al., 2023; Dreke et al., 2020; Fielding-Singh & Dmowska, 2022; Filippini, 2005; Gass & Nichols, 1988; Graham, 2022; Guerin & Ortolan, 2017; Hailes & Goodman, 2023; Harris, 2021; Hayes & Jeffries, 2016; Hester et al., 2023; Hightower, 2017; Jeglic & Winters, 2023; Johnson et al., 2023; Johnson et al., 2021; Jones, 2023; Klein et al., 2023; Knott, 2023; Leedom et al., 2019; Mento et al., 2023; Monterrosa, 2023; Nataraj-Hansen, 2023; Omran & Yousafzai, 2023; Orzeck, 2008; Paul-Mikhail, 2021; Pohlhaus, 2020; Saltmarsh et al., 2022; Sarkis, 2018; Shousha, 2023; Spear, 2023; Stern, 2018; Sweet, 2019; Tanczer et al., 2021; Tarzia & Hegarty, 2023; Tormoen, 2019; Zarr, 2021. |
| Distress; anxiety, depression, etc. | Abramson, 2014; Barton & Whitehead, 1969; Bates, 2020; Calef & Weinshel, 1981; Dickson et al., 2023; Gass & Nichols, 1988; Graham, 2022; H. Stewart, 2019a; Hailes & Goodman, 2023; Hightower, 2017; Klein et al., 2023; Knott, 2023; Li & Samp, 2023; Mento et al., 2023; Miano et al., 2021; Newman et al., 2021; Omran & Yousafzai, 2023; Pohlhaus, 2020; Rodrigues et al., 2021; Stern, 2018; Tormoen, 2019. |
| Blamed themself for the violence | Bates, 2020; Calef & Weinshel, 1981; Dreke et al., 2020; Hegel et al. 2023; Leitão, 2021; Omran & Yousafzai, 2023; Rodrigues et al., 2021; Speed et al., 2021. |
| Isolation | Abramson, 2014; Bates, 2020; Dmitrieva & Glukhova, 2022; Graham, 2022; Graves & Samp, 2021; Guerin & Ortolan, 2017; Hailes & Goodman, 2023; Klein et al., 2023; Knott, 2023; Omran & Yousafzai, 2023; Ridout, 2020; Rodrigues et al., 2021; S. Stewart, 2019; Sarkis, 2018; Spear, 2020; Stern, 2018; Sweet, 2019; Tanczer et al., 2021. |
| Silenced | Bailey, 2020; Dorpat, 1996; Field-Springer et al., 2021; H. Stewart, 2019a; Hailes & Goodman, 2023; Newman et al., 2021; Oddie & Dickey Young, 2020; Peeren et al., 2024; Ridout, 2020; Smith et al., 2023; Spear, 2020; Spear, 2023. |
| Resistance or partial resistance | Hailes & Goodman, 2023; Hightower, 2017; Klein et al., 2023; Leitão, 2021; Li & Samp; Pohlhaus, 2020; Spear, 2020; Tormoen, 2019. |
| Needed to use tools to combat the gaslighting | Bates, 2020; Hailes & Goodman, 2023; Knott, 2023; Leitao, 2021. |

**Supplemental Table 4**. Social Power and Intersectional Vulnerabilities to Gaslighting and Citations

| Intersectional vulnerabilities | Citation that discussed it |
| --- | --- |
| Gender (female) | Abramson, 2014; Alvarez & Miller-Ott, 2022; Badenes-Sastre et al., 2023; Bhatti et al., 2023; Boxall, 2021; Burnett, 2020; Dandelet, 2021; Dickson et al., 2023; Dmitrieva & Glukhova, 2022; Gutowski, 2012; Hailes & Goodman, 2023; Han & Liu, 2023; Johnson et al., 2021; Jones, 2023; Klein et al., 2023; Kroll et al., 2022; Li & Samp, 2023; McGlynn, 2020; McKinnon, 2017; Oliver, 2023; Omran & Yousafzai, 2023; Paul-Mikhail, 2021; Pickens, 2020; Pohlhaus, 2020; S. Stewart, 2019; Sweet, 2019; Tarzia & Hegarty, 2023. |
| Gender (male) | Bates, 2020; Bates & Carthy, 2020 |
| LGBTQ+ | Dorri et al., 2023; Hailes & Goodman, 2023; Klein et al., 2023; Li & Samp, 2023; Sweet, 2019; Treviranus, 2021. |
| Racially marginalized individuals | Alvarez & Miller-Ott, 2022; Badenes-Sastre et al., 2023; Boxall, 2021; Dandelet, 2021; Dorri et al., 2023; Fielding-Singh & Dmowska, 2022; Hailes & Goodman, 2023; Han & Liu, 2023; Jones, 2023; Klein et al., 2023; Li & Samp, 2023; Omran & Yousafzai, 2023; Pickens, 2020; Ridout, 2020; Rodrigues et al., 2021; Sweet, 2019; Tarzia & Hegarty, 2023; Treviranus, 2021. |
| Past victimization | Arabi, 2022; Badenes-Sastre et al., 2023; Boxall, 2021; Dickens, 2006; Gutowski, 2012; H. Stewart, 2019a; Han & Liu, 2023; Hayes & Jeffries, 2016; Hegel et al., 2023; Khattar et al., 2023; Knapp, 2019; Miano et al., 2021; Saltmarsh et al., 2022; Salzberger, 2022; Sweet, 2019; Tormoen, 2019; Treviranus, 2021; Zarr, 2021. |
